# A novel approach to inoperable or recurrent rectal cancer by chemoembolization. A new arrow in our quiver?

**DOI:** 10.18632/oncotarget.9940

**Published:** 2016-06-10

**Authors:** Roberto Bini, Simone Comelli, Renzo Leli, Giacomo Paolo Vaudano, Daniele Savio, Tiziana Viora, Alfredo Addeo

**Affiliations:** ^1^ General Surgery Department, SG Bosco Hospital, Turin, Italy; ^2^ Interventional Radiology- Neuroradiology Department, SG Bosco Hospital, Turin, Italy; ^3^ Oncology Department, Bristol Cancer Center, Bristol, UK

**Keywords:** debiri, rectal cancer, chemoembolization, cancer recurrence

## Abstract

**Purpose:**

Assess the feasibility, safety and efficacy of TACE with irinotecan loaded micro particles (debiri) for the treatment of locally advanced rectal cancer patients.

**Results:**

We assessed the Edmonton Symptom Assessment System (ESAS). The tool is designed to assess nine common symptoms in cancer patients: pain, tiredness, nausea, depression, anxiety, drowsiness, appetite, wellbeing and shortness of breath. The ESAS score was 7 in 10/12 (83%) patients before treatment and 6 in 2/12 (16.5%) patients. After treatment in 6/12 (50%) patients the score dropped to 3; 3/12 (33%) reported 4, 1/12 (8%) reported 2. All patients experienced local control disease with a degree of citoreduction; in 4 cases (33%) we observed outstanding responses with a dramatic reduction in the tumors size which led us to surgical radical resections.

**Materials and methods:**

We run a prospective mono-institutional study where we recruited, 12 non- consecutive patients with histology confirmation of rectal cancer, inoperable and not treatable due to severe comorbidities, or pelvic recurrence/progression after curative treatment, chemotherapy, radiotherapy and/or surgery. Their performance status (PS) ECOG was 2-3. Twelve patients (10 male and 2 female) with a median age 71 (range 56-89) were recruited in the study.

**Conclusions:**

The study has met the primary endpoint and showed encouraging activity. Debiri could be a possible option for locally advanced/inoperable or recurred rectal cancer patients. Further trials are warranted to validate this methodic in early stages.

## INTRODUCTION

Colorectal cancer (CRC) is a leading cause of death, accounting for nearly 10% of all cancer deaths in Western countries. The incidence of rectal cancer in the European Union is ∼35% of the total colorectal cancer incidence, i.e. 15–25/100 000 per year. [1]. The mortality is 4–10/100 000 per year. The overall survival of the patients who do not undergo surgery is rather poorwith less than 25% of the patients alive at 5-year [2].

About 10%–30% of the rectal cancer patients at diagnosis could only receive palliation and approximately 40% of patients, treated with curative intent, develop recurrence that, for the vast majority, cannot be treated with curative intent [3].

Pelvic recurrent rectal cancer remains a challenging clinical problem, and patients generally have a dismal prognosis and a poor quality of life. Goal of palliative care is relieving disease-related symptoms and improving the patient's quality of life [4]. Radical surgical resection of recurrent rectal cancer remains the gold standard; several neoadjuvant treatments have been taken into consideration to increase the resectability rate and to improve long-term survival.

Among them we have been developed an interest in the transarterial chemoembolization (TACE): a minimally invasive therapeutic procedure performed by interventional radiologists that delivers drug intra-arterially in order to stop the vascular supply to the tumour itself and that could be combined with chemotherapy agents, such as Irinotecan [5, 6].

Despite TACE has been widely used in the treatment of liver lesions either secondary or primary, there isn't robust evidence to support such a procedure in the neoadjuvant setting or in locally advanced rectal cancer.

TACE has been performed for many years with a variety of different devices, one of them is the embolic drug-eluting bead (DEB) system where the drug carrier is delivered intra-arterially through catheters that can be positioned close to the main arterial supply that feed particular tumors, causing primarily a physical blockage of the artery in combination with a sustained local delivery of the drug.

We have run a mono institutional study of TACE combined with DEB containing irinotecan (DEBIRI) in locally advanced/inoperable rectal cancer. DEBIRI seems to reduce arterial inflow and drug washout and maximize contact time between the drugs and the tumor cells which results in greater tumour necrosis [7]. The chemotherapy agent, once eluted from the beads, has a very high local concentration which overcomes possible resistance mechanism. The tumour response is likely to be greater than the one with standard arterial infusion.

The primary end points of this study were the feasibility, safety and symptoms control of TACE-DEBIR as treatment of locally advanced rectal cancer patients unsuitable for any other treatment options; the secondary end points were response rate,(RR) which was defined as the volume reduction of target lesions and overall survival (OS).

Alongside the study we issued a quality of evaluation life (QoL) adopting Edmonton scale [8] and EORTC QLQ-C30 [9].

## RESULTS

### Patients and treatment characteristics

Twelve patients (10 males and 2 females) were treated with TACE-Debiri. Eleven had the procedure once and only 1 patient required the treatment twice. (Table 1). The median age was 71( range 56–90): 4 patients had unresectable rectal cancer, not eligible for radiotherapy (RT) or chemotherapy (CHT) due to severe comorbidities, 5 patients presented with isolated pelvic recurrence after surgical resection and conventional treatment including chemotherapy (CHT) and/or radiotherapy (RT) and 3 patients progressed straight after CHT/RT with a locally advanced rectal cancer. All patients presented at the time of the study with at least the one of the following symptoms: obstruction to faecal transit, bleeding and or severe pain. The patients' characteristics are listed in Table 1. Quality of life (QoL) was reported adopting Edmonton scale and EORTC QLQ-C30.

**Table 1:** Patients characteristics

Patients age (years)	PS ECOG	Comorbidities	Clinical Presentation	Follow –up 6 months	Survival (months)
90	2	COPD CAD	RB BO	Stable	10
71	2	Child B CAD with left ventricular hypokinesia	RB BO Pain	Stable disease	5
87	2/3	Essential hypertension, Chronic Atrial Fibrillation, Asthma	RB Pain Tenesmus	Stable	11
70	2	COPD CAD with CCHF	BO Pain Tenesmus Minimal RB	Stable disease	13
42	2	None	RB Pain Tenesmus	Stable disease	18
64	2	CAD COPD	RB BO	Stable disease after surgery	18
62	2	Essential Hypertension	RB BO	No recurrence after surgery Ongoing oncological therapy	15
80	2	CAD COPD	RB BO	Stable disease after surgery	6
73	2	Child A	RB BO Pain	Stable disease after surgery	16
64	2	COPD Child B	BO RB Tenesmus	Stable disease	12
88	2/3	Dilatative Cardiomiopathy Asthma	RB	Stable disease	5
56	2	Essential hypertension	RB Tenesmus	Stable disease	5
70 ± 14					11 ± 5

Legends: CHT = chemotherapy; RT = radiotherapy; RB = rectal bleeding; BO = bowel obstruction;

Legends: CHT= chemotherapy; RT= radiotherapy; RB= rectal bleeding; BO=bowel obstruction;

COPD=Chronic Obstructive Pulmonary Disease; CAD= Coronary artery disease; CCHF=chronic congestive heart failure;

At the time of the procedure 4 out of 12 patients were naïve from any treatment but deemed unfit due to heavy comorbidities, whereas the remaining ones (8/12) had progressed after surgery and/or chemotherapy and radiotherapy and not suitable for any further treatment. (One patient had only radiotherapy prior to Debiri).

### Efficacy

To assess the impact on patients ‘symptoms we adopted the Edmonton Symptom Assessment System (ESAS). This tool has been designed to assess several cancer's symptoms: pain, tiredness, nausea, depression, anxiety, drowsiness, appetite, wellbeing and shortness of breath with the use of an alfa numeric scale that goes from 0, no symptom, to 10, maximum intensity of that symptom.

Overall the ESAS score improved in all score points: the pain score was 7 in 10/12 (83%) patients before treatment and 6 in 2/12 (16.5%) patients. After treatment in 6/12 (50%) patients the pain score dropped to 3 for 6/12 (50% of the patients), 5/12 (41%) reported pain score of 4 and in 1/12 case (9%) the pain reported was 2.

Using EORTC QLQ-C30, the overall mean score decreased from 3 to 2 in 12/12 patients (100%).

**Figure 1:**
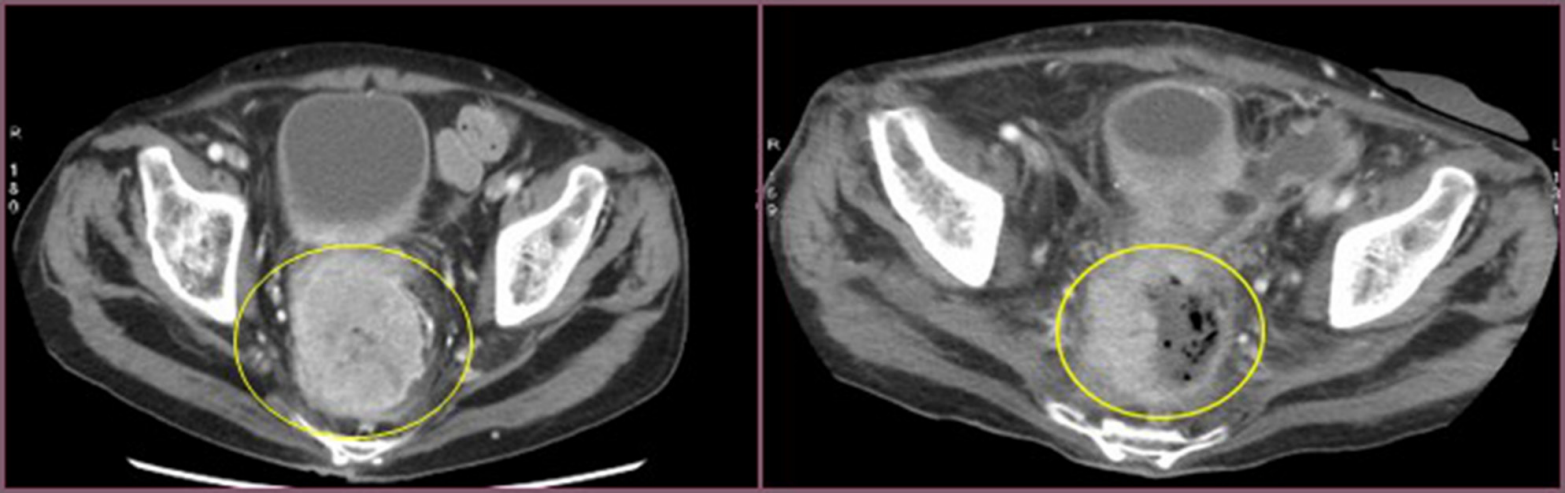
Cross section of contrast enhanced CT scan of the pelvis (venous phase) Left image: into the yellow circle tumor before chemoembolisation. Right image: into the yellow circle the reduction size of the lesion (30 day after treatment).

**Figure 2:**
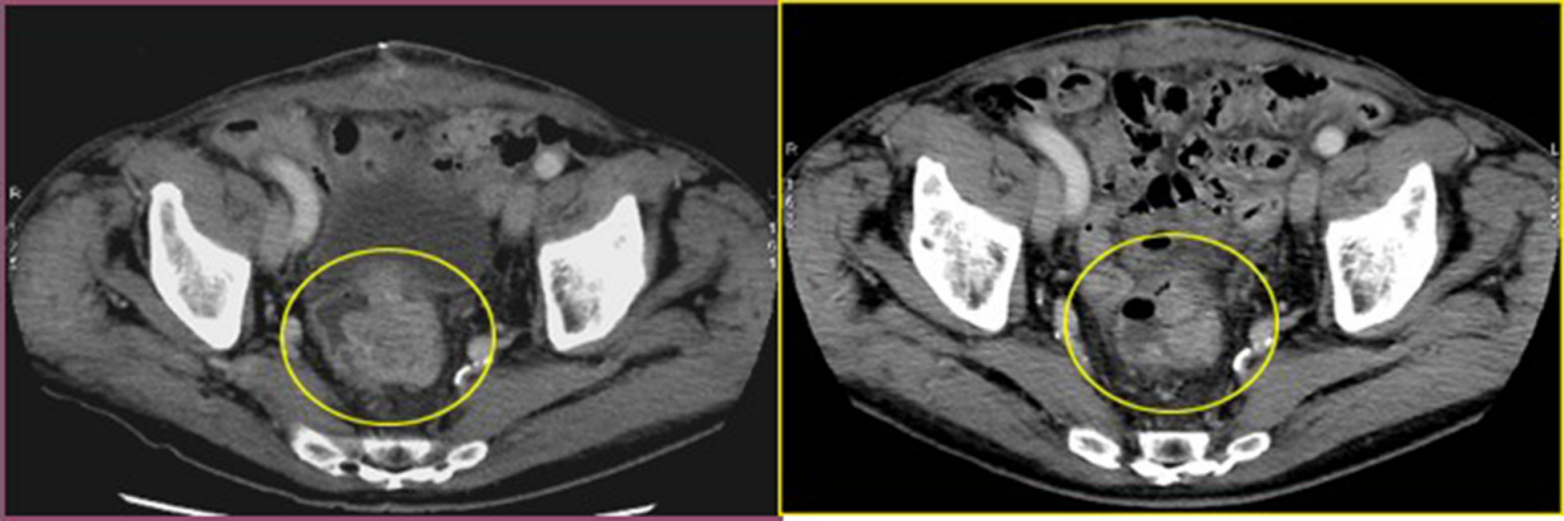
Cross section of contrast enhanced CT scan of the pelvis (venous phase) Left image: into the yellow circle tumor before chemoembolisation; Right image : into the yellow circle the reduction size of the lesion (30 day after treatment).

**Figure 3:**
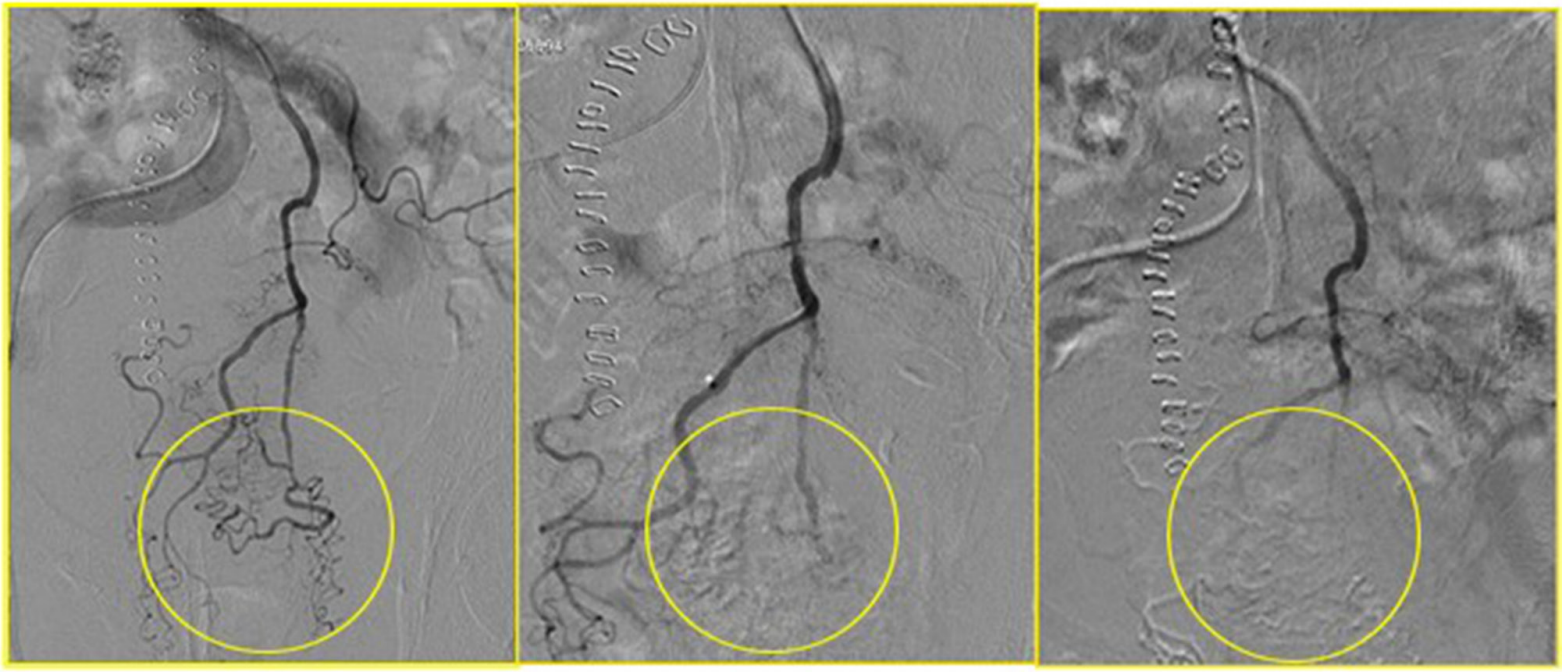
In yellow circle the pregressive disappearing of the tumor vascular bed during the chemoembolisation

All patients experienced local disease control; in 4/12 cases (33%) we observed an outstanding radiological response with a dramatic reduction in the tumor size which led us to radical surgical resection. (Figures 1, 2, 3). Main result of the volume change is summarized in the Table 2. The Only patient who required DEBIRI twice achievied stable disease.

**Table 2:** Radiological evaluation of treatment's response assessed by different criteria

RECIST		mCHOI		MASS	NEW Volume Criteria
% of reduction	Type of response	% of reduction	Type of response	Type of response	% of reduction
37%	PR	41%	PR	FAVORABLE	75%
30%	PR	24%	PR	FAVORABLE	66%
8%	SD	15%	PR	UNDETERMINED	12%
51%	PR	9%	SD	UNDETERMINED	55%
0%	SD	50%	PR	FAVORABLE	63%
0%	SD	53%	PR	FAVORABLE	11%
66%	PR	0%	SD	FAVORABLE	96%
18%	PD	17%	PR	UNDETERMINED	7%
32%	PR	20%	PR	FAVORABLE	24%
18%	PR	1	SD	UNFAVORABLE	5%
31%	PR	18	PR	FAVORABLE	35
28%	PR	20	PR	FAVORABLE	28%
27 ± 16%		22 ± 17			39 ± 26

Legend: PR = Partial response; PD: Progression of the disease; SD = Stable disease.

### Safety

No abnormal laboratory data was present 3 days after the procedure whereas slight increase in WBC, LDH, GOT/GPT and prot C reactive were observed within the first 48 h hours (Table 3). Mild fever lasting one to three days and procedure related pain were observed in10/12 (80%) patients. Persisting muchorrea and tenesmo were reported by 4 patients (33%). The length of In Hospital stay is 3 to 7 days after DEBIRI.

**Table 3:** Patients treatment and adverse event

Patients	Previous Therapy	Number of treat	Adverse Event	DC Beads size	Laboratory data
1	No Surg No CHT or RT	1	Pain Fever	100–300 and 300–500 μm	Increase of: WBC AST/ALT Prot C
2	No Surg No CHT In progression after RT	1	Fever	100–300 and 300–500 μm	Increase of: WBC LDH
3	No Surg Not eligible for CHT and RT	1	Tenesm	100–300 and 300–500 μm	Increase of: WBC LDH AST/ALT
4	No Surg Not eligible for CHT and RT	1	Tenesm	100–300 and 300–500 μm	Increase of: WBC Reactive C Protein
5	Recurrence after Surgery and CH/RT	1	Fever	100–300 and 300–500 μm	Increase of: WBC AST/ALT LDH
6	Recurrence after Surgery No CHT and RT	1	Fever	100–300 and 300–500 μm	Increase of: WBC AST/ALT LDH
7	No surgery in progression after CHT and RT	1	Fever	100–300 and 300–500 μm	Increase of: WBC LDH
8	Recurrence after surgery No CHT or RT	2	Fever Tenesm	100–300 and 300–500 μm	Increase of: WBC AST/ALT Reactive C protein
9	Recurrence after surgery and CHT	1	Fever	100–300 and 300–500 μm	Increase of: WBC AST/ALT
10	Recurrence after surgery	1	Fever	100–300 and 300–500 μm	Increase of: WBC AST/ALT
11	No surg No CHT No RT	1	Fever	100–300 and 300–500 μm	Increase of: Reactive C prot WBC LDH
12	In progression after CHT No RT	1	Fever Tenesm	100–00 and 300–500 μm	Increase of: WBC LDH AST/ALT

Legends: CHT = chemotherapy; RT = radiotherapy; RB = rectal bleeding; BO = bowel obstruction; COPD = Chronic Obstructive Pulmonary Disease; CAD = Coronary artery disease; CCHF = chronic congestive heart failure; WBC = white blood cell; AST = aspartate aminotransferase; ALT = alanine aminotransferase; LDH: lactate dehydrogenase.

The median OS was 12 months (CI 95%: 5 to 18).(Table 4).

**Table 4:** Patients survival and ESAS score (pain)

Patients age (years)	ESAS score PAIN Before/after	Survival (months)	NEW Volume Criteria
90	7/3	10	75%
71	7/3	5	66%
87	7/3	11	12%
70	7/4	13	55%
42	7/3	18	63%
64	6/4	18	11%
62	7/2	15	96%
80	6/3	6	7%
73	7/4	16	24%
64	7/4	12	5%
88	7/4	5	35
56	7/3	5	28%
70 ± 14		11 ± 5	39 ± 26

Legend: ESAS: Edmonton Symptom Assessment System.

## DISCUSSION

Approximately 20% of rectal cancer patients presents with unresectable locally advanced or metastatic disease [10, 11]. Approximately one-half of patients who underwent “curative” resection for localized disease will eventually die of metastatic disease [12]. During that time, patients may present with a variety of disease-related, psychological and physical symptoms: obstruction, bleeding, perforation, pain, weight loss, and fatigue, all affecting and worsening quality of life [4]. Approximately 30 to 50% of these patients die with the locally recurrent tumor the only manifestation of the disease, without experiencing systemic synchronous or metachronous metastases [3].

Angiography for treatment of inoperable and symptomatic patient has been described in the past, with only few articles covering a broad spectrum of diseases including colorectal cancer [13–15].

TACE has been widely used in the past, primarily to treat liver metastases mainly from CRC, but, to the best of our knowledge, this is the first report of Debiri for the treatment of locally advanced rectal cancer unsuitable for further local or systemic treatment.

The primary objectives of the study were feasibility, safety and symptoms control of DEBIRI in rectal cancer patients. The procedure was performed without any major serious event occurred. We run an ESA score to assess the benefit of the treatment (Table 4). Clinical benefit was achieved in all the patients.

The secondary endpoint was response rate and Overal survival. One of the challenges we faced had been identifying objective criteria to assess response in hollow organ like the rectum. We adopted the volume criteria which was obtained by combining 3 different assessing tools: MASS, RECIST and mChoi criteria. According to volume criteria virtually any patient had a degree of response and 4 patients (33%) had a remarkable response which led to radical surgical resection.

Patients with recurrent rectal cancer and poor performance status are known to have a very grim prognosis with a median overall survival of less than 6 months. Albeit the small group of patients, the OS in the study was 12 months (range 5–18) which represents an outstanding result.

A possible weakness of the study was the difficulty to radiologically assess the patients once on follow up. This was mainly due to patients poor performance status. The patients compliance after the procedure was certainly in line with what we'd expect and many of them did not turn up for their follow up CT scan appointment. Although we follow them up remotely, we can't really make any comment on the progression free survival.

The study has met the primary end point and has gone quite beyond our expectations in term of RR and OS in a poor prognostic group of patients.

This preliminary study has certainly proven that the DEBIRI is safe and could effectively relief cancer symptoms. We have also reported exceptional responses which had been archived with minimal adverse event and led to surgical resection in 4 patients.

Further trials, perhaps in an earlier stage, are warranted to further validate the procedure.

## MATERIALS AND METHODS

We have run a prospective mono-institutional study of TACE-Debiri as an exclusive treatment for locally advanced rectal cancer not suitable for any further treatment option. The study was conducted according to the provisions of the Declaration of Helsinki and The good Clinical Practice Guidelines of the international Conference on harmonization. All patients provided written informed consent form to participate in the study.

We enrolled patients who had had previous histology confirmation of rectal cancer, inoperable or not treatable due to severe comorbidities, and/or pelvic recurrence/progression after curative treatment, chemotherapy (CHT), radiotherapy (RT) and/or surgery and poor performance status ECOG (PS ≥ 2).

Each patient underwent an abdominal quadriphasic CT scan (64slices General electric CT scanner) in order to obtain a preliminary arterial mapping of the tumour lesion and chest CT scan to complete the staging.

A diagnostic angiography of superior and inferior mesenteric arteries (SMA and IMA) was performed to define tumour vascularization and feeding arteries, and identify the best target vessels for the treatment and under local anesthesia a 5 F sheath was inserted using a femoral access. Only in a single case, a selective catheterization of ipogastric artery was also performed.

### Debiri procedure

Using a microcatether, under ultrasound fluoroscopic guidance, a solution of 1 up to 2 ml of irinotecan drug-eluting beads (DC-Bead, Biocompatible, UK Ltd) and non-ionic contrast medium was injected into the artery feeding the lesions.

Eleven patients required the procedure only once and 1 patient was treated with Debiri twice with a 30 days gap.

In all cases 100–300 micron irinotecan-charged dc beads were used and in selective cases the procedure was completed injecting 300–500 micron drug- eluting beads.

All the patients had an unenhanced CT study at 24–48 hours after the procedure to exclude periprocedural complications as bleeding, ischemic necrosis or perforation.

A quadriphasic CT study with the same CT scanner was performed one month later as restaging of the treated lesion. The response was read by the treating physician and an independent investigator.

### Imaging and tumour response

Due to the lack of specific criteria to assess solid tumor in hollow viscous we moved to a different diagnostic criteria adopting the 3D volume reduction, size attenuation, morphology and structural change on venous phase CT (MASS) [16], contrast enhanced during mod Choi [17] and RECIST criteria [18].

Using standard criteria, RECIST, was not felt to be accurate enough as this could have had underestimated the overall benefit of the treatment.

Since RECIST was published in 2000, many investigators have confirmed in prospective analyses the validity of substituting unidimensional for bi-dimensional based criteria (With rare exceptions (e.g. mesothelioma)). This makes very difficult to quantify the response of solid tumour in allow viscous. We have overcome this limitation by adopting the concept of steady mass through 3 different worldwide, accepted criteria for solid tumor evaluation: MASS that assesses the change in the attenuation patterns of contrast enhancement on contrast-enhanced CT (CECT); RECIST that is the traditional method of evaluating therapy response based on measurements of long-axis tumor size on axial CT according to the Response Evaluation Criteria in Solid Tumors and mChoi criteria that was initially conceived to assess tumor response in patients with advanced gastrointestinal stromal tumors who received treatment with imatinib, and the criteria were evaluated by computed tomography (CT) scans and validated based on progression-free survival.

Grading of the adverse events was determined using CTCAE version 4. Primary endpoints of the study were feasibility, safety and symptoms control Secondary endpoints were response rate (RR), defined as volume reduction of target lesion and overall survival (OS), defined as time from the last TACE-DEBIRI treatment to death.
